# Blood-Based Brain and Global Biomarker Changes after Combined Hypoxemia and Hemorrhagic Shock in a Rat Model of Penetrating Ballistic-Like Brain Injury

**DOI:** 10.1089/neur.2021.0006

**Published:** 2021-08-12

**Authors:** Xue Li, Kevin Pierre, Zhihui Yang, Lynn Nguyen, Gabrielle Johnson, Juliana Venetucci, Isabel Torres, Brandon Lucke-Wold, Yuan Shi, Angela Boutte, Deborah Shear, Lai Yee Leung, Kevin K.W. Wang

**Affiliations:** ^1^ Program for Neurotrauma, Neuroproteomics, and Biomarkers Research, Department of Emergency Medicine, University of Florida, Gainesville, Florida, USA.; ^2^ Department of Neonatology, Children's Hospital, Chongqing Medical University, Chongqing, China.; ^3^ Brain Trauma Neuroprotection, Center for Military Psychiatry and Neuroscience, Walter Reed Army Institute of Research, Silver Spring, Maryland, USA.; ^4^ Brain Rehabilitation Research Center, Malcom Randall VA Medical Center, Gainesville, Florida, USA.; ^5^ Department of Surgery, Uniformed Services University for the Health Sciences, Bethesda, Maryland, USA.; ^6^ College of Medicine, University of Florida, Gainesville, Florida, USA.; ^7^ Department of Neurosurgery, University of Florida, Gainesville, Florida, USA.

**Keywords:** biomarker, brain injury, global injury markers, organ injury

## Abstract

Penetrating traumatic brain injury (pTBI) often occurs with systemic insults such as hemorrhagic shock (HS) and hypoxemic (HX). This study examines rat models of penetrating ballistic-like brain injury (PBBI) and HX+HS to assess whether the blood levels of brain and systemic response biomarkers phosphorylated neurofilament-heavy protein (pNF-H), neurofilament-light protein (NF-L), αII-spectrin, heat shock protein (HSP70), and high mobility group box 1 protein (HMGB1) can distinguish pTBI from systemic insults and guide in pTBI diagnosis, prognosis, and monitoring. Thirty rats were randomly assigned to sham, PBBI, HS+HX, and PBBI+HS+HX groups. PBBI and sham groups underwent craniotomy with and without probe insertion and balloon expansion, respectively. HX and HS was then simulated by blood withdrawal and fraction of inspired oxygen (FIO_2_) reduction. Biomarker serum concentrations were determined at one (D1) and two (D2) days post-injury with enzyme-linked immunosorbent assay (ELISA) methods. Axonal injury-linked biomarkers pNF-H and NF-L serum levels in PBBI groups were higher than those in sham and HX+HS groups at D1 and D2 post-injury. The same was true for PBBI+HX+HS compared with sham (D2 only for pNF-H) and HX+HS groups. However, pNF-H and NF-L levels in PBBI+HX+HS groups were not different than their PBBI counterparts. At D1, αII-spectrin levels in the HX+HS and PBBI+HS+HX groups were higher than the sham groups. αII-spectrin levels in the HX+HS group were higher than the PBBI group. This suggests HX+HS as the common insult driving αII-spectrin elevations. In conclusion, pNF-H and NF-L may serve as specific serum biomarkers of pTBI in the presence or absence of systemic insults. αII-spectrin may be a sensitive acute biomarker in detecting systemic insults occurring alone or with pTBI.

## Introduction

The incidence and mortality of penetrating traumatic brain injury (pTBI) have more than doubled in the civilian population between 2010 and 2014. Roughly half of these patients suffered severe TBI, with a mortality rate of 43.8%.^1^ These patients often require extensive time in the intensive care unit (ICU) and require extensive rehabilitation services. Unfortunately, limited data exist for pTBI due to its relatively low prevalence.^2^ Further, polytrauma commonly occurs with pTBI after improvised explosive device attacks. Patients who suffered from pTBI and polytrauma face physical, cognitive, and psychological impairments and have a higher mortality rate than those with pTBI alone.^3–5^

A high prevalence of concomitant hypoxemia (HX) and hemorrhagic shock (HS) exists in patients with pTBI and polytrauma. Experimental TBI combined with HX and HS results in worsened neurological outcomes,^6,7^ increased metabolic disturbances,^8^ and neuronal cell death.^9^ For example, our previous study demonstrated the cumulative effects of HS and HX on the physiological, metabolic, and neurobehavioral outcomes in a penetrating ballistic-like brain injury (PBBI) model.^10–12^ Therefore, these systemic insults are often incorporated into the existing pTBI models to increase translational research's clinical relevance.

Brain imaging modalities, including computed tomography (CT) and magnetic resonance imaging (MRI), provide a useful but limited assessment of the injury severity of pTBI. For example, CT exposes patients to radiation and is insensitive for the diagnosis of diffuse axonal injury.^13^ Although MRI better detects axonal injury and subtle neuronal damage, it is not suitable for patients with hemodynamic instability, is more difficult to perform on patients receiving ventilation, and is generally reserved for the subacute stage.^14^ These limitations leave a clinical gap where important data need to be garnered for prognosis and treatment. Readily accessible blood-based biomarkers found within the serum, plasma, or whole blood may play a role in the diagnosis and prognosis of PBBI with or without systemic insults. Additionally, they may provide pathophysiological information of injury severity of pTBI throughout a patient's disease course in the intensive care setting. Further, they may play a role in evaluating the efficacy of treatments of TBI in the setting of polytrauma. The ability to predict mortality and injury severity may facilitate the development of clinical guidelines for managing pTBI.^15^

Several biomarkers are reported to be altered after PBBI, including GFAP (glial fibrillary acidic protein; astrogliosis/astroglial injury),^16–18^ brain-derived neurotrophic factor (BDNF; neurogenesis/neuroprotection),^19^ ubiquitin C-terminal hydrolase-L1 (UCH-L1; neuronal cell body injury),^20^ cathepsin B (CatB; apoptosis/cell necrosis),^21^ and microRNA (miRNA; astrogliosis/inflammation/neurodegeneration).^22,23^

Considering that pTBI and polytrauma often occur concurrently in the setting of emergency medicine, we hypothesized that the use of brain-specific and global injury biomarkers can reliably identify HX+HS and frontal PBBI with and without systemic insults (HX+HS) to diagnose and differentiate combined injury from isolated pTBI. The brain-specific biomarkers of interest include the phosphorylated axonal form of the heavy neurofilament subunit (phosphorylated neurofilament-heavy protein [pNF-H]; axonal injury)^15,24,25^ and the light neurofilament subunit (neurofilament-light protein [NF-L]; axonal injury).^26,27^ The global injury biomarker panel includes the high mobility group box 1 protein (HMGB1; alarmin stress response/cell necrosis)^28–31^ and heat-shock protein (HSP70; organ stress/shock response).^28,32^ Although levels of αII-spectrin and its breakdown products (axonal injury/cell necrosis) have been reported to change in the setting of PBBI, they were also included in this global injury marker panel considering their abundant expression in non-central nervous system (CNS) organs.^15,16,18,33,34^ We also hypothesize that polytrauma will exacerbate brain injury and cause rising levels in brain-specific biomarkers.

## Methods

### Research subjects

Thirty male adult Sprague-Dawley rats (280–320 g, 3 months old; Charles River Labs, Raleigh, NC, USA) were used in these experiments. All protocols involving animal use were reviewed and approved by the Institutional Animal Care and Use Committee of Walter Reed Army Institute of Research. Research was conducted in compliance with the Animal Welfare Act, Guide for the Care and Use of Laboratory Animals (eighth edition, National Research Council), and other federal statutes and regulations. All surgical procedures were performed using aseptic technique. Animals were housed individually under a 12-h light-dark cycle in a temperature-controlled facility accredited by the Association for Assessment and Accreditation of Laboratory Animal Care International. All animals had access to food and water *ad libitum* post-injury.

### PBBI, HX, and HS

Anesthesia was induced in an induction chamber with 3.5% isoflurane delivered in air/oxygen mixture (fraction of inspired oxygen [FIO_2_] = 0.26) and maintained using a nose cone at 1.5% throughout the surgeries. In all animals, the right femoral artery and vein were cannulated using number 2 French tubing (cat. no. BTPU-027; Instech Laboratories) for mean arterial blood pressure (MAP) monitoring and fluid resuscitation, respectively. Using the same tubing, the tail artery was cannulated for inducing HS by withdrawing blood and for blood gas analysis. Partial pressures of oxygen (PaO_2_) and carbon dioxide (PaCO_2_) and pH were measured with a blood gas analyzer (ABL5; Radiometer America Inc., OH, USA) to confirm the hypoxemic condition.

A total of 30 rats were randomly divided into four groups that included sham control (craniotomy only, *n* = 6), PBBI only (*n* = 8), HS+HX only (*n* = 8), and PBBI+HX+HS (*n* = 8) groups. Unilateral (right) PBBI was induced using a simulated ballistic injury device (Mitre Corp., McLean, VA, USA) with a specially designed stainless-steel probe (Popper & Sons Inc., Hyde Park, NY, USA).^35^ The probe was mounted to a stereotaxic arm at an angle of 50 degrees from the vertical axis and 25 degrees counterclockwise from the anteroposterior (A-P) axis. It was then manually inserted through the right frontal cortex of the anesthetized rat via a cranial window (+4.5 mm A-P, +2 mm mediolaterial [M-L] from bregma) to a distance of 12 mm (from dura). The elastic tubing on the probe was inflated by a rapid (40 millisecond) water pressure pulse, forming an elliptical balloon calibrated to 10% of the total rat brain volume causing a temporary intracerebral cavity.

The probe was then gently retracted, and the cranial opening was sealed with sterile bone wax. Transient HX was induced by reducing FIO_2_ to 0.1 (10% oxygen balanced with 90% nitrogen), resulting in a PaO_2_ of less than 40 mm Hg (confirmed by blood gas analysis). Normoxia (FIO_2_ = 0.26) was restored after 30 min of HX. Transient HS was induced by withdrawing blood via the tail arterial catheter using a withdrawal pump (Harvard Apparatus, MA, USA) at a constant rate of 0.25 mL per 100 g/min to reduce the MAP by 5 mm Hg, from 45 mm Hg to 40 mm Hg (monitored via femoral artery catheter). HS was maintained for 30 min. The animals then received fluid resuscitation with lactated Ringer's solution (B. Braun Medical, PA, USA) via the femoral vein catheter. The infusion volume was 3 times the blood volume withdrawn.^36^ In the HX+HS groups, HX started at 5 min after PBBI or sham PBBI, whereas HS started at 5 min after restoring normoxia from HX. The sham control animals received all surgical procedures (including catheterizations and craniotomy) with the exception of the PBBI probe insertion/expansion.

### Serum preparation

Blood samples (2 mL) were collected at terminal end-points (D1 and D2 post-injury) by cardiac puncture using Z/1.3 clotting tubes (Sarstedt, Newton, NC, USA) and were allowed to clot at room temperature for 30 min before centrifugation at 1200*g* for 10 min at 4°C. Serum was transferred to a storage tube and kept in −80°C. All samples were shipped via FedEx priority overnight (on dry ice) to the University of Florida for protein analyses.

### Quantitation of protein biomarker

Dynamic evaluation of brain and global biomarkers was performed at D1 and D2 post-injury in the PBBI model. Serum concentrations of brain and global biomarkers pNF-H (cat no. RD191138300R; Biovendor), NF-L (item 103345; Quanterix), HSP70 (cat no. ab133060; abcam), and inflammation markers HMGB1 (ABIN416082; Antibodies-online.com) and αII-spectrin (cat no. ABIN1572517; Antibodies-online) were determined with sandwich enzyme-linked immunosorbent assays (ELISA) at the University of Florida. Sandwich ELISAs were conducted using standard 96-well, flat-bottom, Nunc Immuno Maxisorp plates (Fisher, Pittsburgh, PA, USA), according to the manufacturer's protocol. Selected capture and detection antibody pairs were used.

Briefly, ELISA plates were passively coated overnight at 4°C with capture antibody, then washed and blocked. Serum samples were added and incubated at room temperature with shaking. After washing, peroxidase-conjugated detection antibody or HRP-conjugated streptavidin were added, which catalyzed the reaction with a colorimetric substrate (TMB; Pierce). The product was quantified by absorbance at 450 nm in a microplate spectrophotometer. Standard curves were generated using recombinant proteins corresponding to the biomarker measured in each assay. Four parameter-fit non-linear regression analyses were applied to determine biomarker quantities.

### Statistical analysis

Statistical analysis was performed using GraphPad Prism (version 9.0.0). Data were presented as mean ± standard error of the mean (SEM). The differences in biomarker concentration among the groups were analyzed with one-way analysis of variance (ANOVA) followed by post hoc comparisons using Tukey's test. The criterion for significance was set at *p* < 0.05.

## Results

### Tissue specificity for biomarkers associated with brain injury and polytrauma

We first conducted omics-based data mining to ascertain specific tissue expression of the five biomarkers of interest. The relative biomarker mRNA expression levels were retrieved from the GeneCards human gene database.^60^ pNF-H (NEFH) and NF-L (NEFL) show high specificity to the CNS (brain, spinal cord). mRNA for HSP70 (HSPA4), and HMGB1 is ubiquitously expressed in all organs and peripheral blood cells.^15,33,34^ Although our data mining show that αII-spectrin (SPTAN1) levels are highest in the CNS, this biomarker is widely distributed among other organs.

We next verified the protein distribution of the same markers in various human organs and cell types. The results are based on mass spectrometry detection of the tryptic peptides derived from the five respective parent proteins shown on the Human Proteome Map portal.^61^ NF-L and NF-H are highly specific and enriched in the brain and spinal cord and are minimally concentrated in other organs or cells. In contrast, HMGB1 and HSP70 show strong protein signals throughout all organs and cells examined. Lastly, αII-spectrin (SPTAN1) protein is enriched in the CNS but is also expressed in other tissues.

### Temporal survey of serum biomarkers

**FIG. 1.**
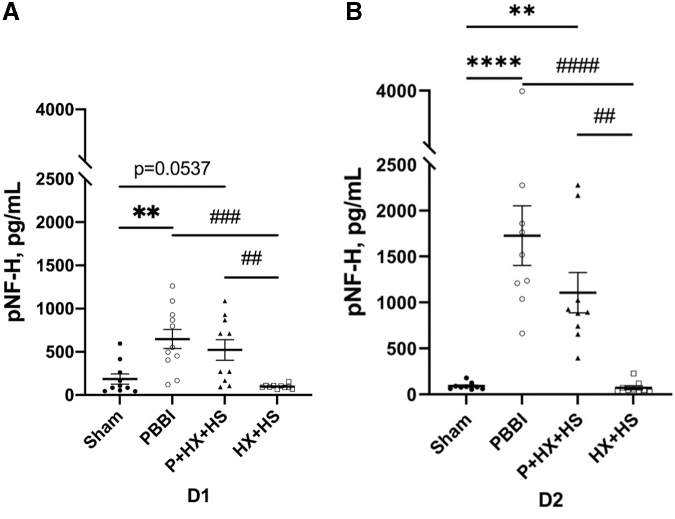
Serum pNF-H levels at D1 **(A)** and D2 **(B)** post-injury. The levels of pNF-H showed a significant increase in PBBI at D1, and in both PBBI and P+HX+HS groups at D2 when compared with sham (**p < 0.01, ****p < 0.0001). The levels of pNF-H significantly increased in PBBI and P+HX+HS groups at D1 and D2 when compared with HS+HX (##*p* < 0.01, ###*p* < 0.001, ####*p* < 0.0001). Biomarker concentration differences were analyzed with ANOVA followed by post hoc comparisons using Tukey's test. Data are presented as mean ± SEM. ANOVA, analysis of variance; D1, one day; D2, two days; HS, hemorrhagic shock; HX, hypoxemia; P, penetrating ballistic-like brain injury; PBBI, penetrating ballistic-like brain injury; pNF-H, phosphorylated neurofilament-heavy protein; SEM, standard error of the mean.

The serum levels of the brain-specific axonal injury marker pNF-H were analyzed at D1 and D2 in all groups (Fig. 1A,B). pNF-H serum levels in animals subjected to PBBI were significantly higher than those in the sham control (*p* < 0.01) and HX+HS (*p* < 0.001) groups at D1. Similarly, pNF-H levels in animals subjected to PBBI were significantly higher than those in the sham control (*p* < 0.0001) and HX+HS (*p* < 0.0001) groups at D2. There was also a significant increase in pNF-H levels in the P+HX+HS groups compared with the HX+HS groups at D1 and D2. There was a non-significant increase at D1 in pNF-H levels in the PBBI group compared with the sham group that reached significance at D2. No significant differences were detected between the HX+HS and sham groups. At D1 and D2 post-injury, pNF-H levels trended lower in the P+HX+HS group compared with those in the PBBI group, but the difference was not significant.

**FIG. 2.**
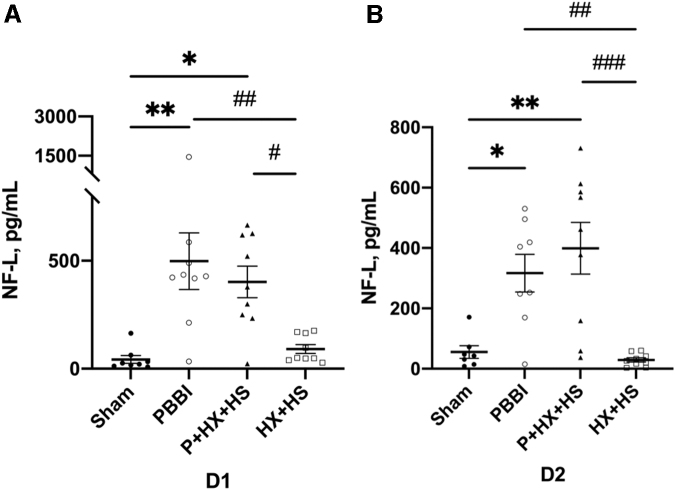
Serum NF-L levels at D1 **(A)** and D2 **(B)** post-injury. The levels of NF-L showed a significant increase in both PBBI and P+HX+HS groups at D1 and D2 when compared with sham (*p < 0.05, **p < 0.01). The levels of pNF-H significantly increased in PBBI and P+HX+HS groups at D1 and D2 when compared with HS+HX (#*p* < 0.05, ##*p* < 0.01, ###*p* < 0.001). Biomarker concentration differences were analyzed with ANOVA followed by post hoc comparisons using Tukey's test. Data are presented as mean ± SEM. ANOVA, analysis of variance; D1, one day; D2, two days; HS, hemorrhagic shock; HX, hypoxemia; NF-L, neurofilament-light protein; P, penetrating ballistic-like brain injury; PBBI, penetrating ballistic-like brain injury; pNF-H, phosphorylated neurofilament-heavy protein; SEM, standard error of the mean.

The serum levels of NF-L were significantly higher in the PBBI (*p* < 0.01) and P+HX+HS (*p* < 0.05) groups at D1 and D2 post-injury. NF-L serum levels were also significantly higher in both PBBI (*p* < 0.01 at D1 and D2) and P+HX+HS (*p* < 0.05 at D1; *p* < 0.001 at D2) groups when compared with HX+HS. There were no statistically significant differences between PBBI and P+HX+HS groups at either day post-injury. (Fig. 2A,B)

**FIG. 3.**
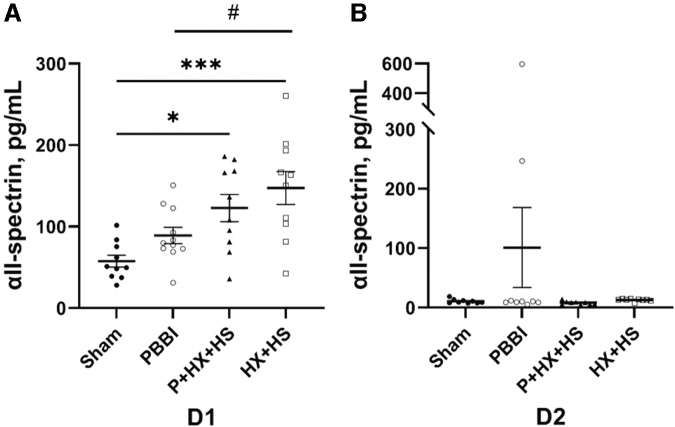
Serum global injury marker αll-spectrin levels at D1 **(A)** and D2 **(B)** post-injury. The levels of αll-spectrin showed a significant increase in HX+HS and P+HX+HS groups (compared with sham *p < 0.05, ***p < 0.001; #*p* < 0.05 compared between PBBI and HX+HS groups). Biomarker concentration differences were analyzed with ANOVA followed by post hoc comparisons using Tukey's test. Data are presented as mean ± SEM. ANOVA, analysis of variance; D1, one day; D2, two days; HS, hemorrhagic shock; HX, hypoxemia; P, penetrating ballistic-like brain injury; PBBI, penetrating ballistic-like brain injury; SEM, standard error of the mean.

αII-spectrin is a major cortical cytoskeleton protein located in the brain and in nucleated cells in most peripheral organs.^15,33,37^ αII-spectrin serum levels in animals subjected to HX+HS were significantly higher than those in the sham control (*p* < 0.001) and PBBI (*p* < 0.05) groups at D1 post-injury (Fig. 3A). Similarly, αII-spectrin levels were significantly higher at D1 in the PBBI+HX+HS groups compared with the sham groups (*p* < 0.05) (Fig. 3A). No significant differences were detected between the PBBI alone and sham groups at D1 post-injury. By D2 post-injury, αII-spectrin levels in all groups returned to sham control levels. (Fig. 3B).

**FIG. 4.**
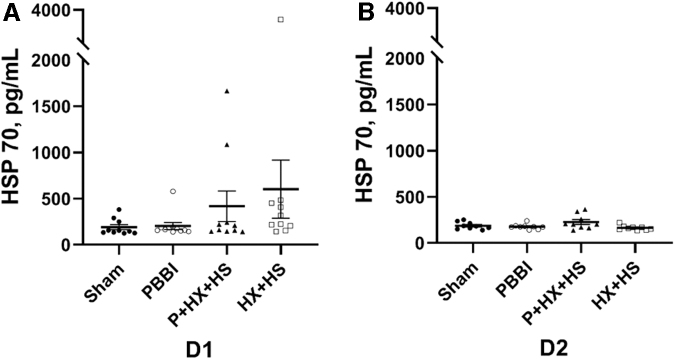
Serum global injury marker HSP70 levels at D1 **(A)** and D2 **(B)** post-injury. There seems to be a trend of HSP70 toward an increase in injured groups than in sham control groups at D1. Otherwise, there are no statistically significant differences between any group at D1 and D2. D1, one day; D2, two days.

Serum levels of global stress response biomarker HSP70 were quantitated at D1 and D2 post-injury (Fig. 4). There was a non-significantly increasing trend of HSP70 levels in the injury groups compared with the sham control group at D1 post-injury (Fig. 4A). At D2 post-injury, the serum levels of HSP70 in all injury groups were comparable to that in the sham control group (Fig. 4B).

**FIG. 5.**
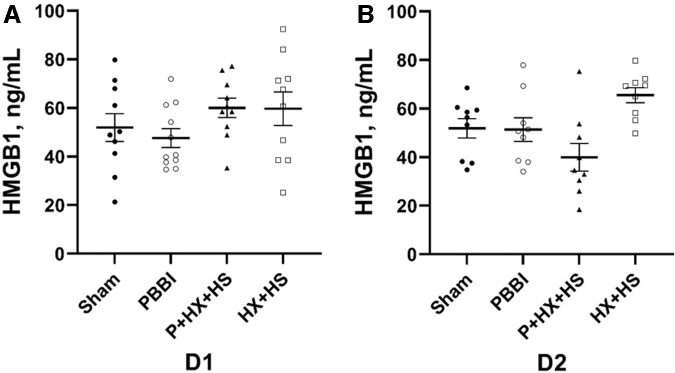
Serum HMGB1 levels at D1 **(A)** and D2 **(B)** post-injury. There are no statistically significant differences between any groups. D1, one day; D2, two days.

Lastly, serum levels of the inflammation biomarker, HMGB1, were quantitated at D1 and D2 post-injury (Fig. 5). No significant differences between groups were detected in the levels of HMGB1 at both time-points.

## Discussion

Our study investigates brain and global injury biomarkers following combined HX and HS (HX+HS) insults in a pTBI model to help differentiate combined injury from isolated TBI. Brain imaging, such as CT and MRI, provides consistent methods to assess the presence and severity of pTBI with or without the influence of polytrauma. However, these imaging techniques remain limited in critical conditions and have unique drawbacks and limitations especially within the acute to subacute period post-injury. Blood-based protein biomarkers may be used to augment the diagnosis, prognosis, and monitoring of pTBI. They may also help monitor evolution secondary injury. Treatments for pTBI are limited but may benefit from a better understanding of acute brain and global protein profiles as well as from identifying sensitive biomarkers to differentiate combined injuries from isolated TBI.

### Brain-enriched biomarkers

Neurofilament (NF) proteins are crucial structural components of the axonal cytoskeleton.^27^ They are composed of three different subunits differentiated by molecular weight (neurofilament-heavy protein [NF-H], neurofilament-medium protein [NF-M], and neurofilament-light protein [NF-L]).^38^ NF-H is heavily phosphorylated (pNF-H).^39^ Several studies suggest that NF proteins may serve as sensitive markers of TBI.^40–47^ pNF-H was reported to be a good predictor of injury severity in patients with mild TBI^48^ and acute spinal cord injury.^49^ In another study using a PBBI model, serum levels of pNF-H at both D1 and D2 post-injury were correlated with the degree of histological injury in a rat model of controlled cortical impact (CCI).^40^ The NF-L isoform is important to consider as it is believed to accumulate more rapidly and have more sustained elevations than pNF-H.^27^ Further, NF-L levels have predicted TBI and patient outcomes.^26^ It is important to consider that both these NF proteins are present in the peripheral nervous system and in muscle. However, they are present in relatively low levels compared with the CNS. Further, there is no evidence that HX+HS or pTBI cause peripheral nerve injury. Our findings were consistent with our hypothesis that pNF-H and NF-L would serve as brain-specific markers for PBBI as pNF-H and NF-L levels in rats subjected to PBBI were elevated compared with the HX+HS and sham control groups.

Whereas we also anticipated an increase in pNF-H in the P+HX+HS group compared with sham, a statistically significant difference was only observed in D2, although it approached significance at D1. This is consistent with a previous study that demonstrated significantly higher levels of pNF-H in a PBBI model at D2 compared with D1 and suggests that pNF-H may serve as a more useful marker at D2.^41^

Because post-traumatic HX and HS have been reported to exacerbate brain damage,^3–5^ it is surprising that these additional insults following TBI did not increase the levels of pNF-H and NF-L. The data may indicate the physiological compensatory response to handling mild to moderate HX and hypotension. Our previous studies demonstrated that the sensitivity capacity may account for the lack of additive effects of hypoxemic and hypotensive insults in animals subjected to 10% PBBI.^10^ This issue may be resolved in future studies by reducing the injury severity in the PBBI model by reducing the volume of balloon expansion to a smaller percentage such as 5% of total brain volume. Further, technical issues, such as from HS, may have contributed to variation in the data, secondary to altered diffusion rates or distributions of biomarkers. It is possible that the HS was not severe enough to aggravate the TBI. It is also possible that the pNF-H and NF-L induced by combined PBBI and HS+HX might increase in a more delayed fashion in our model as seen in other injury types such as delayed cerebral ischemia and diffuse anoxic brain injury. If so, detection of pNF-H at more delayed time-points in future studies might provide novel mechanistic insight. A study in humans demonstrated similar results with no differences in pNF-H levels in patients with TBI with and without polytrauma.^25^

### Global injury biomarkers

αII-spectrin is a cortical cytoskeletal protein with essential roles in neuronal development, synaptic, plasticity, and cytoskeletal remodeling.^50^ It is abundant in neurons but is also found in most other organs.^15,33,34,37^ During cell apoptosis, αII-spectrin is processed into breakdown products of 150i and 120 kDa fragments (SBDP150 and SBDP120) by caspase 3. In cell necrosis, and to a lesser extent during cell apoptosis, αII-spectrin is truncated to 150 and 145 kDa fragments (SBDP150 and SBDP145) by calpain.^50^ Various studies have demonstrated that αII-spectrin may potentially serve as a biomarker in TBI.^51–57^ In the present study, the αII-spectrin ELISA assay detected intact αII-spectrin and its various SBDPs. Our data mining analysis shows that both mRNA levels and protein levels of αII-spectrin (SPTAN1) are abundant in the CNS tissues. Yet, compared with NEFH and NEFL, αII-spectrin levels are also high in various other organs such as the heart, retina, ovary/testis, kidney, and adrenal gland.

Our study suggested that αII-spectrin is a sensitive biomarker for sensitive insults but not PBBI. This was the case as HX+HS served as the common factor for elevated αII-spectrin levels. Further, PBBI levels were not significantly elevated compared with sham in our study despite its high CNS concentration. This is similar to the results from another rat PBBI study reported by Zoltewicz and colleagues^20^ in which plasma SBDP150 was comparable to sham control levels at D1 post-injury. Thus, this serum biomarker's sensitivity to HX+HS and lack of sensitivity and specificity to PBBI may be due to the expression of αII-spectrin in other organs besides the brain and may indicate subtle peripheral organ damage.^15,33,34^ It is important to note that PBBI+HX+HS levels were unexpectantly insignificantly elevated compared with the PBBI group. This may again represent an issue of sensitivity capacity and should be further investigated in future studies by reducing the volume of balloon expansion.

HMGB1 proteins are involved in DNA binding and contribute to chromatin architecture.^58^ They are released from the cell nucleus during cellular necrosis,^31^ serve as an alarmin signaling mediator during inflammatory responses,^30,59^ and may serve as biomarkers in TBI.^29^ HSP70, a protein activated in physiological stress, may also serve as a biomarker in TBI.^32^ No significant changes in these two non-brain organ-specific protein biomarkers^33,34^ were detected. The brain is highly susceptible to HS and HX insults. TBI itself can cause hypoperfusion, decreased oxygen delivery, metabolic dysfunction, and perturbed autoregulation of cerebral blood flow, all which account for the increased vulnerability of the injured brain to hypotensive and/or hypoxemic insults. It is plausible that the durations of HX and HS were not long enough to cause multiple organ dysfunction. In the field, patients can be hypotensive or hypoxemic for significant periods of time prior to the arrival of emergency medical personnel. Further, the HX and HS may have not been severe enough. The primary focus of this study is the effect of combined PBBI and systemic insults on the brain. The impact on other organs requires further analyses in addition to the serum biomarkers studied herein.

### Limitations and future directions

A limitation of this study is the exclusive use of serum; biomarker profiles in cerebrospinal fluid and brain tissue (e.g., microdialysate) could provide a more complete picture. These biofluids are often readily accessible in patients with severe TBI in the ICU setting who often undergo placement of intraventricular catheters.^15^ Further, only acute post-injury time-points (D1 and D2) were examined. More extended time periods could be obtained with clinical samples. Although pNF-H may have a delayed effect following PBBI, further studies should examine subacute to chronic time-points. Considering earlier time-points at 6- or 12-h post-injury for αII-spectrin levels may reveal valuable information considering the return to baseline by D2. The small sample size also serves as a limitation, especially considering the presence of outliers in the data. Finally, reducing PBBI severity may provide a more sensitive platform for evaluating the cumulative effects of combined HX and HS insults. This may be more clinically relevant as well as it would target a representation of patients who are more likely to survive to discharge. Biomarkers profile patterns compared between different severities of PBBI should be considered in further studies. Future research may include behavioral studies to examine biomarkers' role in predicting neurological deficits especially in the context of memory formation and impulsivity.

## Conclusion

This study demonstrates that PBBI increases the serum levels of pNF-H and/or NF-L in rat models. Further, HX and HS, alone or in combination with PBBI, are associated with acute increases in serum αII-spectrin levels, suggesting its utility as a global insult marker that is not brain specific. Therefore, when used together, pNF-H/NF-L and αII-spectrin can serve as promising biomarkers to distinguish PBBI from other insults, to identify systemic insults with or with brain trauma, and to augment clinical monitoring and prognosis of the two conditions.

## Abbreviations Used

ANOVA: analysis of variance
A-P: anteroposterior
BDNF: brain-derived neurotrophic factor
CatB: cathepsin B
CCI: controlled cortical impact
CNS: central nervous system
CT: computed tomography
D1: one day
D2: two days
FIO_2_: fraction of inspired oxygen
GFAP: glial fibrillary acidic protein
HMGB1: high mobility group box 1 protein
HS: hemorrhagic shock
HSP70: heat-shock protein
HX: hypoxemia
ICU: intensive care unit
MAP: mean arterial blood pressure
miRNA: microRNA
M-L: mediolaterial
MRI: magnetic resonance imaging
NF: neurofilament
NF-L: neurofilament-light protein
P: penetrating ballistic-like brain injury
PaCO_2_: partial pressure of carbon dioxide
PaO_2_: partial pressure of oxygen
PBBI: penetrating ballistic-like brain injury
pNF-H: phosphorylated neurofilament-heavy protein
pTBI: penetrating traumatic brain injury
SEM: standard error of the mean
UCH-L1: ubiquitin C-terminal hydrolase-L1

## References

[1] Skarupa, D.J., Khan, M., Hsu, A., Madbak, F.G., Ebler, D.J., Yorkgitis, B., Rahmathulla, G., Alcindor, D., and Joseph, B. (2018). Trends in civilian penetrating brain injury: a review of 26,871 patients. Am. J. Surg. 18, 255–260. 10.1016/j.amjsurg.2018.11.034 30558803

[2] Carney, N., Totten, A.M., O'Reilly, C., Ullman, J.S., Hawryluk, G.W., Bell, M.J., Bratton, S.L., Chesnut, R., Harris, O.A., Kissoon, N., Rubiano, A.M., Shutter, L., Tasker, R.C., Vavilala, M.S., Wilberger, J., Wright, D.W., and Ghajar, J. (2017). Guidelines for the Management of Severe Traumatic Brain Injury, Fourth Edition. Neurosurgery 80, 6–15. 27654000 10.1227/NEU.0000000000001432

[3] Manley, G., Knudson, M.M., Morabito, D., Damron, S., Erickson, V., and Pitts, L. (2001). Hypotension, hypoxia, and head injury: frequency, duration, and consequences. Arch. Surg. (Chicago, IL: 1960) 136, 1118–1123. 11585502 10.1001/archsurg.136.10.1118

[4] McHugh, G.S., Engel, D.C., Butcher, I., Steyerberg, E.W., Lu, J., Mushkudiani, N., Hernandez, A.V., Marmarou, A., Maas, A.I., and Murray, G.D. (2007). Prognostic value of secondary insults in traumatic brain injury: results from the IMPACT study. J. Neurotrauma 24, 287–293. 17375993 10.1089/neu.2006.0031

[5] Peek-Asa, C., McArthur, D., Hovda, D., and Kraus, J. (2001). Early predictors of mortality in penetrating compared with closed brain injury. Brain Inj. 15, 801–810. 11516348 10.1080/02699050010025768

[6] Bramlett, H.M., Dietrich, W.D., and Green, E.J. (1999). Secondary hypoxia following moderate fluid percussion brain injury in rats exacerbates sensorimotor and cognitive deficits. J. Neurotrauma 16, 1035–1047. 10595820 10.1089/neu.1999.16.1035

[7] Shear, D.A., Lu, X.-C.M., Pedersen, R., Wei, G., Chen, Z., Davis, A., Yao, C., Dave, J., and Tortella, F.C. (2011). Severity profile of penetrating ballistic-like brain injury on neurofunctional outcome, blood–brain barrier permeability, and brain edema formation. J. Neurotrauma 28, 2185–2195. 21644814 10.1089/neu.2011.1916

[8] Blanie, A., Vigue, B., Benhamou, D., Duranteau, J., and Geeraerts, T. (2012). The frontal lobe and thalamus have different sensitivities to hypoxia-hypotension after traumatic brain injury: a microdialysis study in rats. J. Neurotrauma 29, 2782–2790. 22860603 10.1089/neu.2012.2381

[9] Bramlett, H.M., Green, E.J., and Dietrich, W.D. (1999). Exacerbation of cortical and hippocampal CA1 damage due to posttraumatic hypoxia following moderate fluid-percussion brain injury in rats. J. Neurosurgery 91, 653–659. 10.3171/jns.1999.91.4.0653 10507388

[10] Leung, L.Y., Deng-Bryant, Y., Shear, D., and Tortella, F. (2015). Combined hypoxemic and hypotensive insults altered physiological responses and neurofunction in a severity-dependent manner following penetrating ballistic-like brain injury in rats. J. Trauma Acute Care Surg. 79, S130–S138. 26406425 10.1097/TA.0000000000000785

[11] Leung, L.Y., Deng-Bryant, Y., Cardiff, K., Winter, M., Tortella, F., and Shear, D. (2016). Neurochemical changes following combined hypoxemia and hemorrhagic shock in a rat model of penetrating ballistic-like brain injury: a microdialysis study. J. Trauma Acute Care Surg. 81, 860–867. 27769083 10.1097/TA.0000000000001206

[12] Leung, L.Y., Wei, G., Shear, D.A., and Tortella, F.C. (2013). The acute effects of hemorrhagic shock on cerebral blood flow, brain tissue oxygen tension, and spreading depolarization following penetrating ballistic-like brain injury. J. Neurotrauma 30, 1288–1298. 23461630 10.1089/neu.2012.2715

[13] Amyot, F., Arciniegas, D.B., Brazaitis, M.P., Curley, K.C., Diaz-Arrastia, R., Gandjbakhche, A., Herscovitch, P., Hinds, S.R., 2nd, Manley, G.T., Pacifico, A., Razumovsky, A., Riley, J., Salzer, W., Shih, R., Smirniotopoulos, J.G., and Stocker, D. (2015). A review of the effectiveness of neuroimaging modalities for the detection of traumatic brain injury. J. Neurotrauma 32, 1693–1721. 26176603 10.1089/neu.2013.3306 PMC4651019

[14] Lee, B., and Newberg, A. (2005). Neuroimaging in traumatic brain imaging. NeuroRx 2, 372–383. 15897957 10.1602/neurorx.2.2.372 PMC1064998

[15] Wang, K.K., Yang, Z., Zhu, T., Shi, Y., Rubenstein, R., Tyndall, J.A., and Manley, G.T. (2018). An update on diagnostic and prognostic biomarkers for traumatic brain injury. Expert Rev. Mol. Diagn. 18, 165–180. 29338452 10.1080/14737159.2018.1428089 PMC6359936

[16] DeDominicis, K.E., Hwang, H., Cartagena, C.M., Shear, D.A., and Boutte, A.M. (2018). Cerebrospinal fluid biomarkers are associated with glial fibrillary acidic protein and alphaii-spectrin breakdown products in brain tissues following penetrating ballistic-like brain injury in rats. Front. Neurol. 9, 490. 30022967 10.3389/fneur.2018.00490 PMC6039567

[17] Mondello, S., Shear, D.A., Bramlett, H.M., Dixon, C.E., Schmid, K.E., Dietrich, W.D., Wang, K.K.W., Hayes, R.L., Glushakova, O., Catania, M., Richieri, S.P., Povlishock, J.T., Tortella, F.C., and Kochanek, P.M. (2016). Insight into pre-clinical models of traumatic brain injury using circulating brain damage biomarkers: operation brain trauma therapy. J. Neurotrauma 33, 595–605. 26671651 10.1089/neu.2015.4132

[18] Boutté, A.M., Deng-Bryant, Y., Johnson, D., Tortella, F.C., Dave, J.R., Shear, D.A., and Schmid, K.E. (2015). Serum glial fibrillary acidic protein predicts tissue glial fibrillary acidic protein break-down products and therapeutic efficacy after penetrating ballistic-like brain injury. J. Neurotrauma 33, 147–156. 25789543 10.1089/neu.2014.3672

[19] Madathil, S.K., Deng-Bryant, Y., Wilfred, B.S., Leung, L.Y., Gilsdorf, J.S., and Shear, D.A. (2017). Alterations in brain-derived neurotrophic factor and insulin-like growth factor-1 protein levels after penetrating ballistic-like brain injury in rats. J. Trauma Acute Care Surg. 83, S16–S24. 28628600 10.1097/TA.0000000000001471

[20] Zoltewicz, J.S., Mondello, S., Yang, B., Newsom, K.J., Kobeissy, F., Yao, C., Lu, X.C., Dave, J.R., Shear, D.A., Schmid, K., Rivera, V., Cram, T., Seaney, J., Zhang, Z., Wang, K.K., Hayes, R.L., and Tortella, F.C. (2013). Biomarkers track damage after graded injury severity in a rat model of penetrating brain injury. J. Neurotrauma 30, 1161–1169. 23409698 10.1089/neu.2012.2762

[21] Boutté, A.M., Hook, V., Thangavelu, B., Sarkis, G.A., Abbatiello, B.N., Hook, G., Jacobsen, J.S., Robertson, C.S., Gilsdorf, J., Yang, Z., Wang, K.K.W., and Shear, D.A. (2020). Penetrating traumatic brain injury triggers dysregulation of cathepsin B protein levels independent of cysteine protease activity in brain and cerebral spinal fluid. J. Neurotrauma 37, 1574–1586. 31973644 10.1089/neu.2019.6537 PMC7307674

[22] Johnson, D., Cartagena, C.M., Tortella, F.C., Dave, J.R., Schmid, K.E., and Boutte, A.M. (2017). Acute and subacute microRNA dysregulation is associated with cytokine responses in the rodent model of penetrating ballistic-like brain injury. J. Trauma Acute Care Surg. 83, S145–S149. 28452880 10.1097/TA.0000000000001475

[23] Thangavelu, B., Wilfred, B.S., Johnson, D., Gilsdorf, J.S., Shear, D.A., and Boutté, A.M. (2020). Penetrating ballistic-like brain injury leads to microRNA dysregulation, BACE1 upregulation, and amyloid precursor protein loss in lesioned rat brain tissues. Front. Neurosci. 14, 915–915. 33071724 10.3389/fnins.2020.00915 PMC7530327

[24] Žurek, J., and Fedora, M. (2012). The usefulness of S100B, NSE, GFAP, NF-H, secretagogin and Hsp70 as a predictive biomarker of outcome in children with traumatic brain injury. Acta Neurochir. (Wien) 154, 93–103. 21976236 10.1007/s00701-011-1175-2

[25] Shibahashi, K., Doi, T., Tanaka, S., Hoda, H., Chikuda, H., Sawada, Y., Takasu, Y., Chiba, K., Nozaki, T., Hamabe, Y., and Ogata, T. (2016). The serum phosphorylated neurofilament heavy subunit as a predictive marker for outcome in adult patients after traumatic brain injury. J. Neurotrauma 33, 1826–1833. 27098610 10.1089/neu.2015.4237

[26] Gao, W., Zhang, Z., Lv, X., Wu, Q., Yan, J., Mao, G., and Xing, W. (2020). Neurofilament light chain level in traumatic brain injury: a system review and meta-analysis. Medicine (Baltimore) 99, e22363–e22363. 32957411 10.1097/MD.0000000000022363 PMC7505327

[27] Siedler, D.G., Chuah, M.I., Kirkcaldie, M.T., Vickers, J.C., and King, A.E. (2014). Diffuse axonal injury in brain trauma: insights from alterations in neurofilaments. Front. Cell. Neurosci. 8, 429. 25565963 10.3389/fncel.2014.00429 PMC4269130

[28] Gan, Z.S., Stein, S.C., Swanson, R., Guan, S., Garcia, L., Mehta, D., and Smith, D.H. (2019). Blood biomarkers for traumatic brain injury: a quantitative assessment of diagnostic and prognostic accuracy. Front. Neurol. 10, 446–446. 31105646 10.3389/fneur.2019.00446 PMC6498532

[29] Paudel, Y.N., Shaikh, M.F., Chakraborti, A., Kumari, Y., Aledo-Serrano, Á., Aleksovska, K., Alvim, M.K.M., and Othman, I. (2018). HMGB1: a common biomarker and potential target for TBI, neuroinflammation, epilepsy, and cognitive dysfunction. Front. Neurosci. 12, 628–628. 30271319 10.3389/fnins.2018.00628 PMC6142787

[30] Rider, P., Voronov, E., Dinarello, C.A., Apte, R.N., and Cohen, I. (2017). Alarmins: feel the stress. J. Immunol. 198, 1395. 28167650 10.4049/jimmunol.1601342

[31] Raucci, A., Palumbo, R., and Bianchi, M.E. (2007). HMGB1: a signal of necrosis. Autoimmunity 40, 285–289. 17516211 10.1080/08916930701356978

[32] Zhang, J., Tao, D.Q., Zhao, H., and Yin, Z.Y. (2012). Expression of Hsp70 and caspase-3 in rabbits after severe traumatic brain injury. Chin. J. Traumatol. 15, 338–341. 23186922

[33] Kim, M.S., Pinto, S.M., Getnet, D., Nirujogi, R.S., Manda, S.S., Chaerkady, R., Madugundu, A.K., Kelkar, D.S., Isserlin, R., Jain, S., Thomas, J.K., Muthusamy, B., Leal-Rojas, P., Kumar, P., Sahasrabuddhe, N.A., Balakrishnan, L., Advani, J., George, B., Renuse, S., Selvan, L.D., Patil, A.H., Nanjappa, V., Radhakrishnan, A., Prasad, S., Subbannayya, T., Raju, R., Kumar, M., Sreenivasamurthy, S.K., Marimuthu, A., Sathe, G.J., Chavan, S., Datta, K.K., Subbannayya, Y., Sahu, A., Yelamanchi, S.D., Jayaram, S., Rajagopalan, P., Sharma, J., Murthy, K.R., Syed, N., Goel, R., Khan, A.A., Ahmad, S., Dey, G., Mudgal, K., Chatterjee, A., Huang, T.C., Zhong, J., Wu, X., Shaw, P.G., Freed, D., Zahari, M.S., Mukherjee, K.K., Shankar, S., Mahadevan, A., Lam, H., Mitchell, C.J., Shankar, S.K., Satishchandra, P., Schroeder, J.T., Sirdeshmukh, R., Maitra, A., Leach, S.D., Drake, C.G., Halushka, M.K., Prasad, T.S., Hruban, R.H., Kerr, C.L., Bader, G.D., Iacobuzio-Donahue, C.A., Gowda, H., and Pandey, A. (2014). A draft map of the human proteome. Nature 509, 575–581. 24870542 10.1038/nature13302 PMC4403737

[34] Stelzer, G., Rosen, N., Plaschkes, I., Zimmerman, S., Twik, M., Fishilevich, S., Stein, T.I., Nudel, R., Lieder, I., Mazor, Y., Kaplan, S., Dahary, D., Warshawsky, D., Guan-Golan, Y., Kohn, A., Rappaport, N., Safran, M., and Lancet, D. (2016). The GeneCards suite: from gene data mining to disease genome sequence analyses. Curr. Protoc. Bioinformatics 54, 1.30.31–31.30.33. 27322403 10.1002/cpbi.5

[35] Williams, A.J., Hartings, J.A., Lu, X.C., Rolli, M.L., Dave, J.R., and Tortella, F.C. (2005). Characterization of a new rat model of penetrating ballistic brain injury. J. Neurotrauma 22, 313–331. 15716636 10.1089/neu.2005.22.313

[36] Schutz, C., Stover, J.F., Thompson, H.J., Hoover, R.C., Morales, D.M., Schouten, J.W., McMillan, A., Soltesz, K., Motta, M., Spangler, Z., Neugebauer, E., and McIntosh, T.K. (2006). Acute, transient hemorrhagic hypotension does not aggravate structural damage or neurologic motor deficits but delays the long-term cognitive recovery following mild to moderate traumatic brain injury. Crit. Care Med. 34, 492–501. 16424733 10.1097/01.ccm.0000198326.32049.7f PMC2377280

[37] Zhang, Z., Larner, S.F., Liu, M.C., Zheng, W., Hayes, R.L., and Wang, K.K. (2009). Multiple alphaII-spectrin breakdown products distinguish calpain and caspase dominated necrotic and apoptotic cell death pathways. Apoptosis 14, 1289–1298. 19771521 10.1007/s10495-009-0405-z

[38] Dautigny, A., Pham-Dinh, D., Roussel, C., Felix, J.M., Nussbaum, J.L., and Jollès, P. (1988). The large neurofilament subunit (NF-H) of the rat: cDNA cloning and in situ detection. Biochem. Biophys. Res. Commun. 154, 1099–1106. 2457365 10.1016/0006-291x(88)90254-9

[39] Julien, J.P., and Mushynski, W.E. (1982). Multiple phosphorylation sites in mammalian neurofilament polypeptides. J. Biol. Chem. 257, 10467–10470. 7202005

[40] Anderson, K.J., Scheff, S.W., Miller, K.M., Roberts, K.N., Gilmer, L.K., Yang, C., and Shaw, G. (2008). The phosphorylated axonal form of the neurofilament subunit NF-H (pNF-H) as a blood biomarker of traumatic brain injury. J. Neurotrauma 25, 1079–1085. 18729720 10.1089/neu.2007.0488 PMC2820728

[41] Yang, Z., Zhu, T., Mondello, S., Akel, M., Wong, A.T., Kothari, I.M., Lin, F., Shear, D.A., Gilsdorf, J.S., Leung, L.Y., Bramlett, H.M., Dixon, C.E., Dietrich, W.D., Hayes, R.L., Povlishock, J.T., Tortella, F.C., Kochanek, P.M., and Wang, K.K.W. (2018). Serum-based phospho-neurofilament-heavy protein as theranostic biomarker in three models of traumatic brain injury: an Operation Brain Trauma Therapy study. J. Neurotrauma 36, 348–359. 29987972 10.1089/neu.2017.5586

[42] Inoue, R., Sumitani, M., Ogata, T., Chikuda, H., Matsubara, T., Kato, S., Shimojo, N., Uchida, K., and Yamada, Y. (2017). Direct evidence of central nervous system axonal damage in patients with postoperative delirium: a preliminary study of pNF-H as a promising serum biomarker. Neurosci. Lett. 653, 39–44. 28504118 10.1016/j.neulet.2017.05.023

[43] Li, Y., Zhang, L., Kallakuri, S., Cohen, A., and Cavanaugh, J.M. (2015). Correlation of mechanical impact responses and biomarker levels: a new model for biomarker evaluation in TBI. J. Neurol. Sci. 359, 280–286. 26671128 10.1016/j.jns.2015.08.035

[44] Martínez-Morillo, E., Childs, C., García, B.P., Álvarez Menéndez, F.V., Romaschin, A.D., Cervellin, G., Lippi, G., and Diamandis, E.P. (2015). Neurofilament medium polypeptide (NFM) protein concentration is increased in CSF and serum samples from patients with brain injury. Clin. Chem. Lab. Med. 53, 1575–1584. 25720124 10.1515/cclm-2014-0908

[45] Neselius, S., Zetterberg, H., Blennow, K., Marcusson, J., and Brisby, H. (2013). Increased CSF levels of phosphorylated neurofilament heavy protein following bout in amateur boxers. PLoS One 8, e81249. 24260563 10.1371/journal.pone.0081249 PMC3829937

[46] Oliver, J.M., Jones, M.T., Kirk, K.M., Gable, D.A., Repshas, J.T., Johnson, T.A., Andréasson, U., Norgren, N., Blennow, K., and Zetterberg, H. (2016). Serum neurofilament light in american football athletes over the course of a season. J. Neurotrauma 33, 1784–1789. 26700106 10.1089/neu.2015.4295

[47] Al Nimer, F., Thelin, E., Nyström, H., Dring, A.M., Svenningsson, A., Piehl, F., Nelson, D.W., and Bellander, B.-M. (2015). Comparative assessment of the prognostic value of biomarkers in traumatic brain injury reveals an independent role for serum levels of neurofilament light. PloS one 10, e0132177–e0132177. 26136237 10.1371/journal.pone.0132177 PMC4489843

[48] Gatson, J.W., Barillas, J., Hynan, L.S., Diaz-Arrastia, R., Wolf, S.E., and Minei, J.P. (2014). Detection of neurofilament-H in serum as a diagnostic tool to predict injury severity in patients who have suffered mild traumatic brain injury. J. Neurosurg. 121, 1232–1238. 25192482 10.3171/2014.7.JNS132474

[49] Singh, A., Kumar, V., Ali, S., Mahdi, A.A., and Srivastava, R.N. (2017). Phosphorylated neurofilament heavy: a potential blood biomarker to evaluate the severity of acute spinal cord injuries in adults. Int. J. Crit. Illn. Inj. Sci. 7, 212–217. 29291173 10.4103/IJCIIS.IJCIIS_73_16 PMC5737062

[50] Yan, X.X., Jeromin, A. and Jeromin, A. (2012). Spectrin Breakdown Products (SBDPs) as Potential Biomarkers for Neurodegenerative Diseases. Curr. Transl. Geriatr. Exp. Gerontol. Rep. 1, 85–93. 23710421 10.1007/s13670-012-0009-2 PMC3661686

[51] Pike, B.R., Flint, J., Dutta, S., Johnson, E., Wang, K.K., and Hayes, R.L. (2001). Accumulation of non-erythroid alpha II-spectrin and calpain-cleaved alpha II-spectrin breakdown products in cerebrospinal fluid after traumatic brain injury in rats. J. Neurochem. 78, 1297–1306. 11579138 10.1046/j.1471-4159.2001.00510.x

[52] Mondello, S., Robicsek, S.A., Gabrielli, A., Brophy, G.M., Papa, L., Tepas, J., Robertson, C., Buki, A., Scharf, D., Jixiang, M., Akinyi, L., Muller, U., Wang, K.K., and Hayes, R.L. (2010). αII-spectrin breakdown products (SBDPs): diagnosis and outcome in severe traumatic brain injury patients. J. Neurotrauma 27, 1203–1213. 20408766 10.1089/neu.2010.1278 PMC2942904

[53] Pike, B.R., Flint, J., Dave, J.R., Lu, X.C., Wang, K.K., Tortella, F.C., and Hayes, R.L. (2004). Accumulation of calpain and caspase-3 proteolytic fragments of brain-derived alphaII-spectrin in cerebral spinal fluid after middle cerebral artery occlusion in rats. J. Cereb. Blood Flow Metab. 24, 98–106. 14688621 10.1097/01.WCB.0000098520.11962.37

[54] Pineda, J.A., Lewis, S.B., Valadka, A.B., Papa, L., Hannay, H.J., Heaton, S.C., Demery, J.A., Liu, M.C., Aikman, J.M., Akle, V., Brophy, G.M., Tepas, J.J., Wang, K.K., Robertson, C.S., and Hayes, R.L. (2007). Clinical significance of alphaII-spectrin breakdown products in cerebrospinal fluid after severe traumatic brain injury. J. Neurotrauma 24, 354–366. 17375999 10.1089/neu.2006.003789

[55] Brophy, G.M., Pineda, J.A., Papa, L., Lewis, S.B., Valadka, A.B., Hannay, H.J., Heaton, S.C., Demery, J.A., Liu, M.C., Tepas, J.J., 3rd, Gabrielli, A., Robicsek, S., Wang, K.K., Robertson, C.S., and Hayes, R.L. (2009). alphaII-Spectrin breakdown product cerebrospinal fluid exposure metrics suggest differences in cellular injury mechanisms after severe traumatic brain injury. J. Neurotrauma 26, 471–479. 19206997 10.1089/neu.2008.0657 PMC2848834

[56] Berger, R.P., Hayes, R.L., Richichi, R., Beers, S.R., and Wang, K.K. (2012). Serum concentrations of ubiquitin C-terminal hydrolase-L1 and αII-spectrin breakdown product 145 kDa correlate with outcome after pediatric TBI. J. Neurotrauma 29, 162–167. 22022780 10.1089/neu.2011.1989 PMC3253308

[57] Siman, R., Giovannone, N., Hanten, G., Wilde, E.A., McCauley, S.R., Hunter, J.V., Li, X., Levin, H.S., and Smith, D.H. (2013). Evidence that the blood biomarker SNTF predicts brain imaging changes and persistent cognitive dysfunction in mild TBI patients. Front. Neurol. 4, 190. 24302918 10.3389/fneur.2013.00190 PMC3831148

[58] Baxevanis, A.D., and Landsman, D. (1995). The HMG-1 box protein family: classification and functional relationships. Nucleic Acids Res. 23, 1604–1613. 7784217 10.1093/nar/23.9.1604 PMC306904

[59] Okuma, Y., Liu, K., Wake, H., Zhang, J., Maruo, T., Date, I., Yoshino, T., Ohtsuka, A., Otani, N., Tomura, S., Shima, K., Yamamoto, Y., Yamamoto, H., Takahashi, H.K., Mori, S., and Nishibori, M. (2012). Anti-high mobility group box-1 antibody therapy for traumatic brain injury. Ann. Neurol. 72, 373–384. 22915134 10.1002/ana.23602

[60] Safran, M., Dalah, I., Alexander, J., Rosen, N., Iny Stein, T., Shmoish, M., Nativ, N., Bahir, I., Doniger, T., Krug, H., Sirota-Madi, A., Olender, T., Golan, Y., Stelzer, G., Harel, A., and Lancet, D. (2010). GeneCards Version 3: The human gene integrator. Database 10.1093/database/baq020. (Last accessed May 1, 2021). PMC2938269 20689021

[61] Kim, M. S., Pinto, S. M., Getnet, D., Nirujogi, R. S., Manda, S. S., Chaerkady, R., Madugundu, A. K., Kelkar, D. S., Isserlin, R., Jain, S., Thomas, J. K., Muthusamy, B., Leal-Rojas, P., Kumar, P., Sahasrabuddhe, N. A., Balakrishnan, L., Advani, J., George, B., Renuse, S., Selvan, L. D., and Pandey, A. (2014). A draft map of the human proteome. Nature, 509, 575–581. 24870542 10.1038/nature13302 PMC4403737

